# Synergistically Augmenting Cancer Immunotherapy by Physical Manipulation of Pyroptosis Induction

**DOI:** 10.1007/s43657-023-00140-y

**Published:** 2024-06-22

**Authors:** Chenyang Zhao, Tingting Zheng, Run Wang, Xiaona Lin, Zhengming Hu, Zhuofei Zhao, Zhifei Dai, Desheng Sun

**Affiliations:** 1 Department of Ultrasonography, Peking University Shenzhen Hospital, Shenzhen, 518036 Guangdong China; 2 Department of Biomedical Engineering, College of Future Technology, National Biomedical Imaging Centre, Peking University, Beijing, 100871 China

**Keywords:** Programmed cell death, Pyroptosis, Cancer, Physical therapy, Cancer immunotherapy

## Abstract

Pyroptosis is a newly recognized type of programmed cell death mediated by the gasdermin family and caspase. It is characterized by the formation of inflammasomes and the following inflammatory responses. Recent studies have elucidated the value of pyroptosis induction in cancer treatment. The inflammatory cytokines produced during pyroptosis can trigger immune responses to suppress malignancy. Physical approaches for cancer treatment, including radiotherapy, light-based techniques (photodynamic and photothermal therapy), ultrasound-based techniques (sonodynamic therapy and focused ultrasound), and electricity-based techniques (irreversible electroporation and radiofrequency ablation), are effective in clinical application. Recent studies have reported that pyroptosis is involved in the treatment process of physical approaches. Manipulating pyroptosis using physical approaches can be utilized in combating cancer, according to recent studies. Pyroptosis-triggered immunotherapy can be combined with the original anti-tumor methods to achieve a synergistic therapy and improve the therapeutic effect. Studies have also revealed that enhancing pyroptosis may increase the sensitivity of cancer cells to some physical approaches. Herein, we present a comprehensive review of the literature focusing on the associations between pyroptosis and various physical approaches for cancer and its underlying mechanisms. We also discussed the role of pyroptosis-triggered immunotherapy in the treatment process of physical manipulation.

## Introduction

These years have witnessed the establishment of new forms of programmed cell death (PCD) distinct from apoptosis, including necroptosis, pyroptosis, and ferroptosis (Bertheloot et al. 2021; Hirschhorn and Stockwell 2019). Researchers have extensively explored these novel types of PCD as potential cancer treatments due to the growing issue of tumor resistance to apoptosis induction (Chen et al. 2021; Gong et al. 2019; Tan et al. 2021). Pyroptosis is one of those PCDs that has drawn much attention since it can trigger an intense inflammatory response, making it a viable target for cancer interventions.

Pyroptosis, known as the inflammatory process of cell death, is characterized by the involvement of the gasdermin (GSDM) protein family and inflammasomes. Typically, cellular recognition of cellular recognition of danger-associated molecular patterns (DAMPs) and pathogen-associated molecular patterns (PAMPs) activates pyroptosis-mediated GSDM proteins, which subsequently triggers the production of inflammasomes and cellular damage. Inflammatory cytokines are released during the pyroptotic process.

Pyroptosis is thought to have a direct relationship with the mechanisms of various diseases, such as infections, autoimmune diseases, cardiovascular diseases, and degenerative diseases (Jia et al. 2019; Pezuk 2019; Shen et al. 2018; Song et al. 2017). The relationship between pyroptosis and malignancies is complicated. According to recent findings, pyroptosis may play a dual role in malignancies. On the one hand, pyroptosis-related signaling pathways and inflammatory mediators are closely associated with tumorigenesis and chemotherapy resistance (Zhou and Fang 2019). On the other hand, studies have demonstrated that the robust inflammatory response induced by pyroptosis of tumor cells may result in remarkable tumor regression (Tan et al. 2021). Therefore, pyroptosis-triggered immunotherapy has drawn a lot of attention from researchers. The inflammatory effectors of pyroptosis are immunogenic and can activate immune responses that are beneficial in treating cancer. Hence, manipulating pyroptosis and pyroptosis-triggered immunotherapy may offer novel clinical strategies for overcoming cancer.

Regulating pyroptosis has been combined with conventional cancer treatments recently due to its ability to inhibit tumor proliferation and migration. Studies have demonstrated that certain anti-tumor drugs, such as ivermectin (Draganov et al. 2015), berberin (Chu et al. 2016), metformin (Zheng et al. 2020), and anthocyanin (Yue et al. 2019), can inhibit tumor growth by inducing caspase-dependent pyroptosis. Moreover, novel therapeutic molecules designed to induce pyroptosis have also been reported, such as *B-Raf proto-oncogene*, *serine/threonine kinase* (*BRAF*) (Erkes et al. 2020) and *Mitogen-activated protein kinase kinase* (*MEK*) inhibitors for treating melanoma and small-molecule inhibitors for lung cancers targeting *Kirsten rat sarcoma viral oncogene homolog* (*KRAS*), *Epidermal growth factor receptor* (*EGFR*), or *Anaplastic lymphoma kinase* (*ALK*) (Lu et al. 2018). Therapeutic agents that can cause an apoptosis-to-pyroptosis switch have also been developed to overcome apoptosis resistance of cancer cells (Chen et al. 2019). Pyroptosis-regulating agents may also augment cancer immunotherapy by promoting the expression of gasdermin-D (GSDMD) (Xiong et al. 2021).

Physical approaches, which employ physical components for treatment purposes, including radiation, light, heat, ultrasonic waves, and electricity, play a critical role in cancer treatment. Radiotherapy (RT) is one of the most widely used physical treatments clinically. Other physical approaches, including radiofrequency ablation, focused ultrasound, microwave ablation, and laser ablation, have also been applied clinically in recent years. Furthermore, nanotechnology-based physical approaches, including photodynamic therapy (PDT), photothermal therapy (PTT), and sonodynamic therapy (SDT), have been extensively investigated for cancer treatment in this decade. PDT and SDT employ the reaction of photosensitizers (PS) and oxygen under light or ultrasonic wave illumination to generate reactive oxygen species (ROS) for killing cancer cells. PTT utilizes laser-activated PSs to convert light into heat for destroying cancers. These novel physical approaches, characterized by their non-invasiveness and low toxicity, have shown promising results in cancer treatment.

**Fig. 1:**
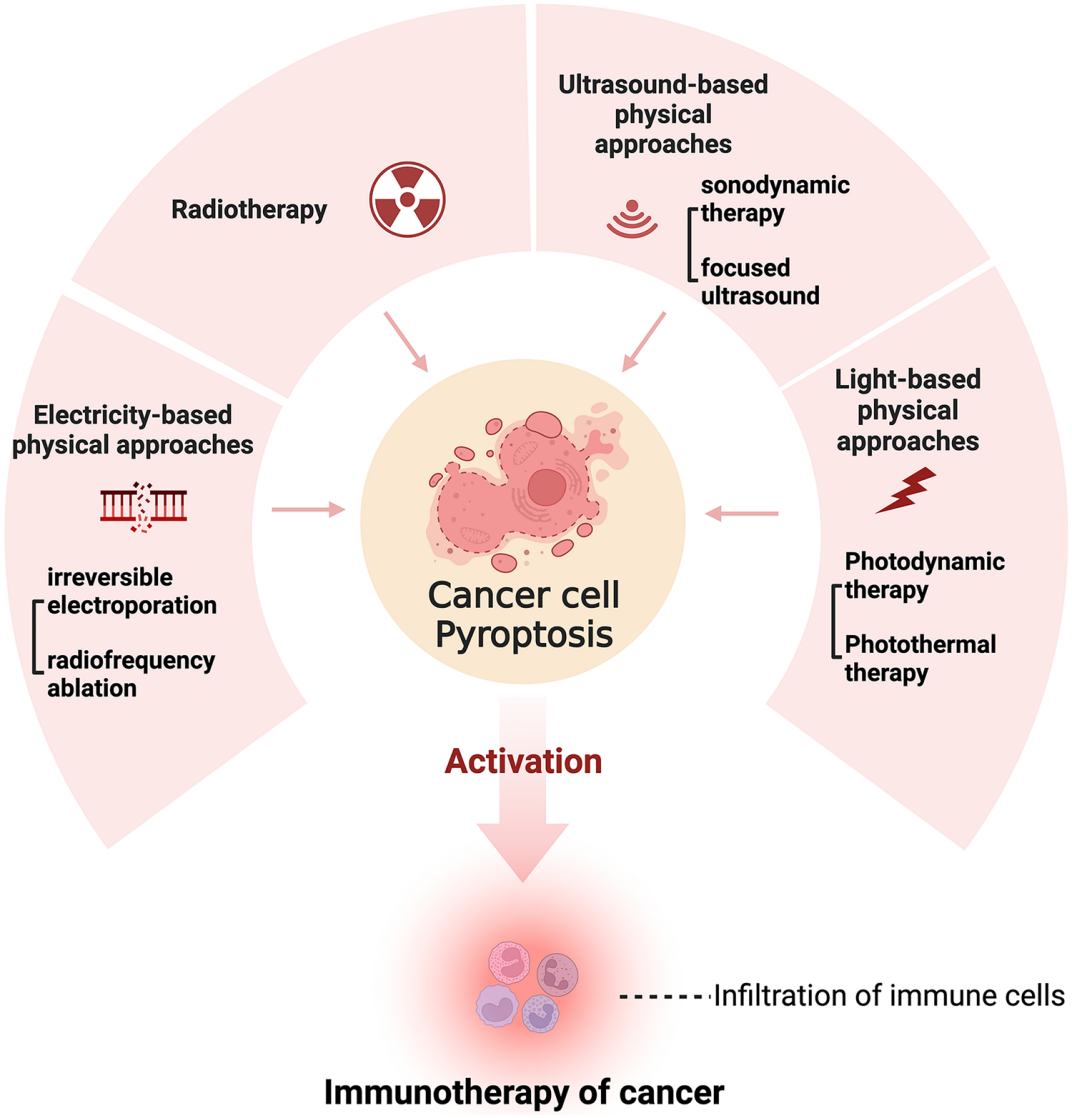
Overview of pyroptosis and physical approaches for cancer treatment. Cell pyroptosis is involved in the treatment process of various physical approaches for cancer treatment, including light-based, ultrasound-based, electricity-based, and radiation-based approaches. The activation of immunotherapy by pyroptosis may further enhance the treatment efficacy of these therapies. The figure was created with BioRender.com

Recent studies suggest that pyroptosis may also have a role in the treatment process of physical approaches in addition to previously reported pyroptosis-induced therapeutic agents. The physical manipulation of pyroptosis and pyroptosis-triggered immunotherapy may offer a novel strategy to inhibit tumor growth and combat drug resistance. However, the tissue damage caused by pyroptosis during physical treatment is a concern. Therefore, a comprehensive summary of physical approaches-related pyroptosis and pyroptosis-induced immunotherapy is required, as this information has not yet been described in previous literature about pyroptosis. This review focused on the associations between pyroptosis and physical approaches for cancer and summarized current developments in this area. The overall view of the study is illustrated in Fig. 1. We discussed the researches about the involvement of pyroptosis in various physical approaches, including light-based approaches, ultrasound-based approaches, radiotherapy, and electricity-based approaches. We aimed to explore the potential of manipulating pyroptosis and pyroptosis-triggered immunotherapy in physical approaches for cancer treatment and to understand pyroptosis in oncology better. To the best of our knowledge, this is the first review of physical approaches-related pyroptosis in cancer treatment.

## Mechanism of Pyroptosis and Pyroptosis-Triggered Cancer Immunotherapy

Pyroptosis was initially discovered in 1990 as a unique form of pathogen-infected macrophage suicide. Pyroptosis was recognized as a distinct death process, although it shared some characteristics with apoptosis (Chen et al. 1996; Zychlinsky et al. 1992). Later in 2001, D'Souza and Heitman (2001) defined this form of inflammation-triggering programmed cell death as pyroptosis, highlighting its inflammatory nature, which set it apart from the non-inflammatory apoptosis. Table 1 lists the similarities and differences between pyroptosis and apoptosis. Pyroptosis shares similar characteristics to apoptosis, such as membrane blebbing, chromosome condensation, and caspase-3 involvement (Chen et al. 2016; Kerr et al. 1972; Kurokawa and Kornbluth 2009; Rudel and Bokoch 1997). However, cytoplasm flattening, pore formation, membrane and nucleus integrity in morphology, caspase-1, 4, 5, 11 involvement, gasdermin family mediation, and inflammatory cytokines releasing are distinctive characteristics of pyroptosis (Aachoui et al. 2013; Broz and Dixit 2016). Both pyroptosis and apoptosi can be stained by Annexin V. In contrast, 7-Aminoactinomycin D (7-AAD) and propidine iodide (PI) staining can only be positive in pyroptosis, which could be used to identify cell pyroptosis (Chen et al. 2016; Tajima et al. 2019).

**Table 1:** Comparisons of cell pyroptosis and apoptosis in cellular morphology and mechanism

Characteristics	Pyroptosis	Apoptosis
Morphology		
Cell swelling	√	×
Cell shrinking	×	√
Pore formation	√	×
Membrane integrity	×	√
Nucleus integrity	√	×
Chromatin condensation	√	√
Mechanism		
Inflammation induction	√	×
Caspase involvement	Caspase-1/3/4/5/6/8/9/11	Caspase-3/6/8
Gasdermin mediation	√	×
PARP mediation	×	√
Staining	Annexin V/ TUNEL/ 7-AAD / PI / EtBr	Annexin V/ TUNEL

PARP: poly-ADP-ribose polymerase; TUNEL: Terminal-deoxynucleoitidyl Transferase Mediated Nick End Labeling; 7-AAD: 7-Aminoactinomycin D; PI: propidine iodide, EtBr: Ethidium bromide

**Fig. 2:**
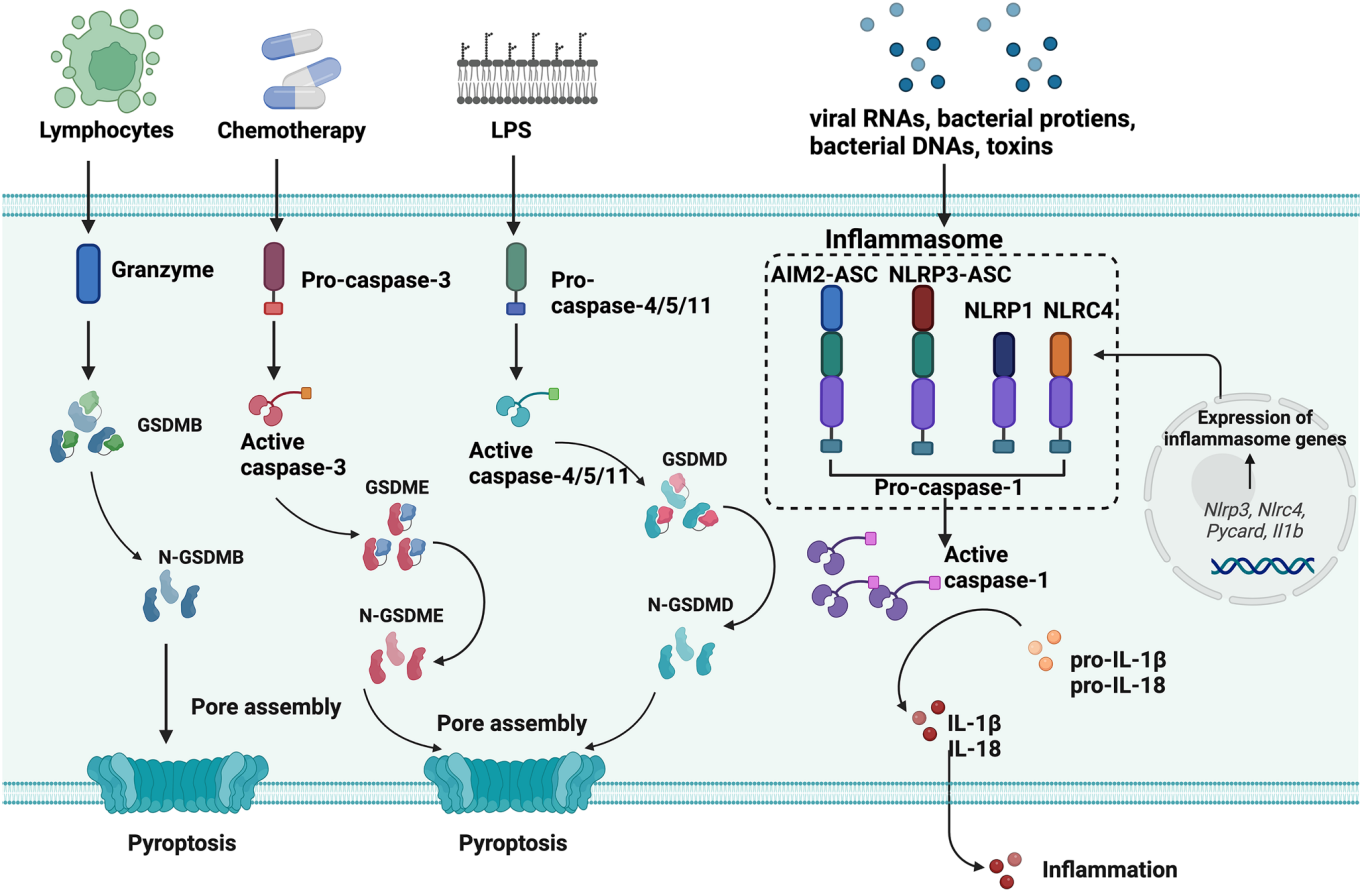
Schematic of the molecular mechanism of pyroptosis. The canonical pathway of pyroptosis is mediated by caspase-1 and GSDMD. Sensor proteins, including NOD-like receptor family pyrin domain-containing protein 1 (NLRP1), NOD-like receptor family pyrin domain-containing protein 3 (NLRP3), NOD-like receptor family caspase recruitment domain (CARD) domain-containing protein 4 (NLRC4), and Absent in melanoma 2 (AIM2), activate inflammasome assembly in response to exogenous or endogenous stimulations. Caspase-1 is activated from pro-caspase 1 by the inflammasome adaptor protein apoptosis-associated speck-like protein containing CARD (ASC). GSDMD is cleaved by active caspase-1, releasing its N-terminus, which contributes to the pore formation of cells. Inflammatory cytokines, IL-1β and IL-18, are also activated by caspase-1 simultaneously. The non-canonical pathway is the activation of caspase-4/5/11 and cleavage of GSDMD when binding with cytosolic lipopolysaccharide (LPS). The caspase-3/GSDME pathway of pyroptosis can occur in chemotherapy. And granzyme/GSDMB pathway is found in lymphocyte-derived pyroptosis. The figure was created with BioRender.com

Figure 2 depicts the mechanism of pyroptosis. The gasdermin family, constituted by gasdermin A (GSDMA), gasdermin B (GSDMB), gasdermin C (GSDMC), gasdermin D (GSDMD), gasdermin E (GSDME), and Deafness, autosomal recessive 59 protein (DFNB59), are the major effectors of pyroptosis. GSMDA-E proteins possess two domains, the pore-forming domain (PFD) in the N-terminal that induces pyroptosis and the repressor domain (RD) in the C-terminal that inhibits PFD function (Aglietti and Dueber 2017). After the cleavage of gasdermin proteins, PFD is separated from RD, causing cellular pore formation and subsequent inflammatory responses of cells (Ding et al. 2016). Gasdermin proteins are also linked to the genesis of various diseases and play a part in pyroptosis-related cancer treatment (Wang et al. 2017).

Pyroptosis is typically either inflammasome-dependent, involving canonical and non-canonical pathways, or inflammasome-independent, involving caspase-3 and granzymes proteases. GSDMD-mediated inflammasome formation and interleukin-1 beta (IL-1β) and interleukin-18 (IL-18) releasing are critical features of the canonical pathway of cell pyroptosis. Inflammasome sensors, including Nucleotide-binding oligomerization domain (NOD)-like receptor family pyrin domain-containing protein 1 (NLRP1), NOD-like receptor family pyrin domain-containing protein 3 (NLRP3), NOD-like receptor family caspase recruitment domain (CARD) domain-containing protein 4 (NLRC4), Absent in melanoma 2 (AIM2), and pyrin, contain a caspase recruitment domain CARD for pro-caspase-1 recruitment and inflammasome formation. Upon the recognition of PAMPs and DAMPs by inflammasome sensors, pro-caspase-1 was activated, followed by the inflammasome assembly (Broz and Dixit 2016; Lamkanfi and Dixit 2017). GSDMD, the major effector of the canonical pathway of pyroptosis, is cleaved by active caspase, causing the release of pore-forming gasdermin-N domain (Shi et al. 2015). Pro-inflammatory cytokines, IL-1β and IL-18, are generated directly through the cleavage of active caspase-1. The pyroptotic cells can release high mobility group protein B1 (HMGB1) and adenosine triphosphate (ATP) after cellular rupture of pyroptosis. The secretion of inflammatory cytokines IL-1β, IL-18, HMGB1 and ATP triggers the inflammatory response, which is a crucial step in the pyroptotic process.

Human caspase-4/5 (mouse caspase-11) activates the non-canonical pyroptosis pathway (Shi et al. 2014). The caspases cleaved GSDMD after interacting with lipopolysaccharide (LPS) by its N-terminal CARD, causing pyroptosis (Kayagaki et al. 2015). In the non-canonical pyroptosis, IL-1β and IL-18 cannot be directly produced by caspase-4/5 but are matured through the NLRP3/caspase-1 pathway (Zanoni et al. 2016).

Caspase-3/GSDME and caspase-8/GSDMC mediated pyroptosis are independent pathways of pyroptosis without forming canonical or non-canonical inflammasomes, which were detected in chemotherapy-induced pyroptosis (Wang et al. 2017). The Granzyme-B/GSDME and Granzyme-A/GSDMB driven pyroptosis have also been discovered in natural killer cells and lymphocytes, which may further enhance the immune response to cancer (Liu et al. 2020; Zhang et al. 2020; Zhou et al. 2020).

Pyroptosis triggers immune responses in cancer cells via two major pathways: the secretion of inflammatory cytokines IL-1β and IL-18 activated by caspase and the release of HMGB1 and ATP following pyroptotic cellular rupture (Volchuk et al. 2020). Both IL-1β and IL-18 are crucial factors in innate and adaptive immune responses. IL-1β promotes the maturation of cytotoxic T lymphocytes and the antigen recognition of T cells. NK cells and Th1 cells, which have IL-18 receptors, can be activated by IL-18. IL-18 can also form a positive loop with interferon-γ (IFN-γ), which exerts a series of immune activities for cancer suppression, including the activation of cytotoxic T lymphocytes (CTLs), the production of granzyme B, and the inhibition of immunosuppressive cytokines (Mantovani et al. 2019; Nakanishi 2018). Pyroptosis increases ATP levels in the cancer microenvironment, facilitating macrophage migration and dendritic cell (DCs) recruitment (Wang et al. 2013b). HMGB1 triggers innate immune responses by activating macrophages and secreting tumor necrosis factor (TNF) via its interaction with Toll-like receptor 4. Additionally, HMGB1 causes infiltration of CTLs and upregulation of major histocompatibility complex-II on DCs (Erkes et al. 2020; Yang et al. 2010). Pyroptosis can trigger immune responses through its inflammatory products, which can be a complementary therapy to the initial treatments.

## Light-Based Physical Approaches and Pyroptosis

Light irradiation-based physical approaches have been developed for cancer treatment, including laser ablation and nanomaterial-based physical–chemical methods, PDT and PTT. Because the involvement of pyroptosis in laser ablation has not been reported by previous literature, this section mainly focused on PDT- and PTT- related pyroptosis.

### Photo-pyroptosis: Triggering Synergic Treatment of Cell Death and Immunotherapy via ROS Production

**Fig. 3:**
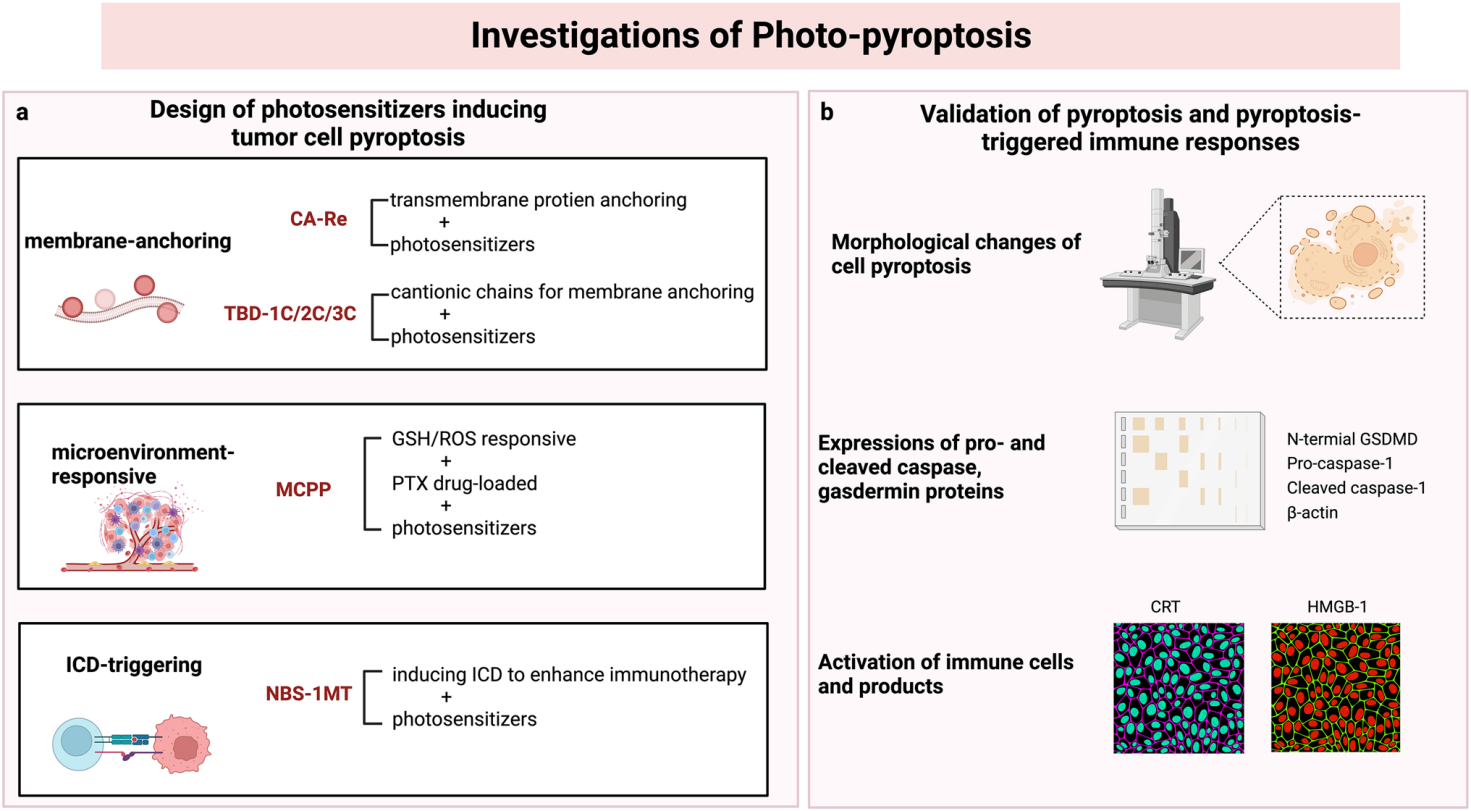
Illustrations of the investigations exploring photo-pyroptosis. **a** The design of photosensitizers inducing tumor cell pyroptosis; **b** Validation of pyroptosis and pyroptosis-triggered immune responses. Photo-pyroptosis photosensitizers are endowed with versatile functions, including carbonic anhydrase IX (CAIX) anchored rhenium (I)-based photosensitizer with membrane anchoring photosensitizers with aggregation-induced emission characteristics, smart tumor microenvironmental ROS/glutathione (GSH) responsive nano-prodrug, and photosensitizers with the indoleamine 2,3-dioxygenase inhibitor for the inhibition of immunosuppressive cells (Lu et al. 2021; Su et al. 2022; Wu et al. 2021; Xiao et al. 2021). The existence of pyroptosis in the PDT process was validated by detecting pyroptotic cellular changes and pyroptosis-related effectors. And the activation of immune responses was also verified by observing the immune cells and products. The figure was created with BioRender.com

PDT is a widely investigated nanotechnology-based physical approach for treating cancer that utilizes photosensitizers to produce cell-killing ROS under laser irradiation and oxygen supply. It is widely acknowledged that PDT induces apoptosis of cancer cells to achieve its treatment purpose, which hardly forms immune responses and may cause drug resistance (Chen et al. 2018). Pyroptosis, however, has been proven to be involved in PDT and to be able to elicit anti-tumor immune responses, which has a synergistic therapeutic effect with PDT. It has been revealed that cellular oxidative stress could facilitate the release of immunogenetic DAMPs during PDT (Huang et al. 2019). And ROS, the critical effector molecule of PDT, can activate NLPR3, an inflammasome sensor crucial to the canonical pathway of pyroptosis (Lu et al. 2021). Researchers have coined the term “photo-pyroptosis” to describe PDT-induced pyroptosis and its synergistic immunotherapy because of the close relationship between pyroptosis and PDT. Some studies have designed photo-pyroptosis photosensitizers with versatile functions for cancer treatment. These studies established the participation of pyroptosis in PDT and the activation of immune responses triggered by photo-pyroptosis, as depicted in Fig. 3 (Lu et al. 2021; Su et al. 2022; Wu et al. 2021; Xiao et al. 2021).

Su et al. (2022) developed a metal-based photo-pyroptosis complex to combat primary malignancy and distant metastasis. The complex, named as CA-Re, was constituted by rhenium (I)-based photosensitizers that could anchor on carbonic anhydrase IX (CAIX), a transmembrane protein overexpressed on tumor cells. It could exert a photodynamic reaction and produce ROS on cell membranes in situ by anchoring the transmembrane proteins. Morphological changes of pyroptosis were observed in the CA-Re-treated cells, such as swollen cells, pore formation on the plasma membranes, and intact nuclei. Researchers also detected an increased level of gasdermin D N-terminal domain (GSDMD-N) and cleaved caspase-1 within the cancer cells, suggesting the existence of the caspase-1/GSDMD pathway of pyroptosis. CA-Re significantly suppressed primary and metastatic tumors. Furthermore, the activation of immune cells, including DCs and CTLs, was also observed following PDT treatment. The results indicated that CA-Re could be fixed on cell membranes and induce GSDMD-mediated pyroptosis by producing ROS upon laser irradiation. The secretion of inflammatory cytokines during the pyroptotic process evoked adaptive immune responses. The pyroptosis-induced immunotherapy contributed to the elimination of distant tumors, thus forming synergistic therapy with PDT to suppress cancer.

Similarly, Wu et al. (2021) developed a series of membrane-anchoring photosensitizers with strong PDT efficacy and pyroptosis-inducing capability. By releasing ROS on membranes in situ, these membrane-anchoring photosensitizers activate caspase-1/GSDMD-mediated pyroptosis, which results in the production of pro-inflammatory cytokines and immunological responses. The study discovered that human breast cancer cells exposed to photosensitizers and laser irradiation expressed significant levels of cleaved GSDMD and caspase-1. Additionally, the ability of photosensitizers to adhere to membranes increased their potential to trigger pyroptosis. The study also found that pyroptosis and apoptosis coexisted in the PDT-treated cancer cells, and that pyroptosis could become dominant by modifying the charge property of the photosensitizers (Wu et al. 2021).

Apart from the caspase-1/GSDMD pathway for pyroptosis, as mentioned above, PDT might also induce pyroptosis through the caspase-3/GSDME axis. Li et al. (2021) revealed that PDT could trigger cell pyroptosis via the caspase-8/caspase-3/GSDME pathway by inhibiting pyruvate kinase M2 (PKM2) in esophageal squamous cell carcinoma. They found that cancer cells had increased levels of PKM2, gasdermin E N-terminal domain (GSDME-N) and cleaved-caspase-3. The study also found that GSDME-silencing cells displayed reduced morphological changes of pyroptosis. And blocking caspase-8 and caspase-3 could attenuate PDT-triggered pyroptosis.

A highlight of photo-pyroptosis treatment was the combination with immunotherapy. Lu et al. (2021) developed a photo-pyroptosis agent by binding the photosensitizer N-Bromosuccinimide (NBS) with the indoleamine 2,3-dioxygenase (IDO) inhibitor, 1-methyltryptophan (1-MT), to achieve PDT-induced pyroptosis and CTL promotion simultaneously. IDO serves as a tumor-promoting enzyme by degrading tryptophan and increasing kynurenine, activating regulatory T cells and constraining CTLs (Munn and Mellor 2016). 1-MT could reverse the immunosuppressive effects of IDO and assist in CTL activation in the tumor microenvironment. Inflammatory cytokines released by PDT-triggered pyroptosis lead to the maturation of DCs and infiltration of CTL. This study provides a typical example of photo-pyroptosis and pyroptosis-triggered immunotherapy for enhancing the anti-tumor effect. The immunotherapy could also assist in the inhibition of distant metastatic lesions.

Due to its immune-triggering property, photo-pyroptosis could also be combined with immune checkpoint blockade (ICB) for cancer treatment. Xiao et al. (2021) developed a ROS-/glutathione (GSH)- responsive prodrug loaded with paclitaxel and purpurin 18 photosensitizers, named as microenvironmental ROS/GSH dual-responsive nano prodrug (MCPP), which could induce pyroptosis under laser irradiation and be further utilized as an immune adjuvant of anti-programmed death-1 (anti-PD-1) therapy to prevent tumor recurrence. MCPP released drugs in the tumor microenvironment with high ROS and GSH concentrations. Under laser irradiation, MCPP achieved the synergistic effect of GSDME-dependent photo-pyroptosis and chemotherapy. The MCPP-triggered pyroptosis elicited an anti-tumor immune response as an immune adjuvant for ICB therapy. The study successfully combined MCPP treatment with programmed death-1 (PD-1) blockage therapy to inhibit tumor growth and improve the prognosis of the animal models.

**Fig. 4:**
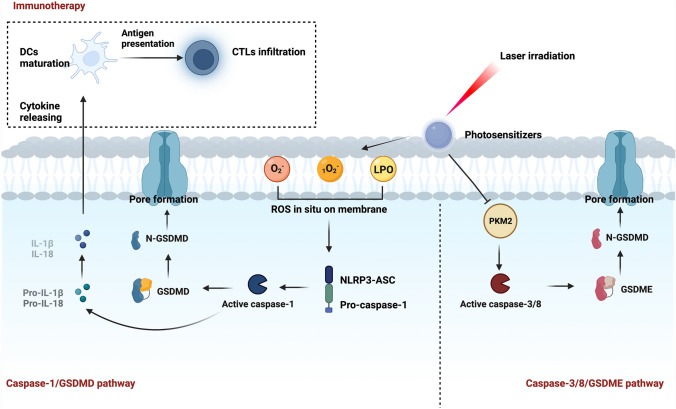
Schematic of photo-pyroptosis. It is reported that photo-pyroptosis can be induced through the caspase-1/GSDMD pathway and caspase-3/8/GSDME pathway. Reactive oxygen species (ROS) produced in the photodynamic therapy (PDT) process is the main stimulant of the caspase-1/GSDMD pathway. The photosensitizers designed by Su et al. (2022) and Wu et al. (2021) had the membrane-anchoring ability and could generate ROS in situ on cellular membranes. ROS was subsequently recognized by NOD-like receptor family pyrin domain-containing protein 3(NLRP3)-apoptosis-associated speck-like protein containing CARD (ASC), activating the caspase-1/GSDMD pathway of pyroptosis. Inflammatory cytokines were released simultaneously and triggered an anti-tumor immune response. PDT could also start caspase-3/8 by inhibiting pyruvate kinase M2 (PKM2), thus activating the caspase-3/8/GSDME pathway of pyroptosis (Li et al. 2021)

Generally, PDT photosensitizers are known to trigger apoptosis of tumor cells. Based on the studies mentioned above, PDT could also inhibit tumor growth via the caspase-1/GSDMD pathway and caspase-3/caspase-8/GSDME pathway of pyroptosis, as shown in Fig. 4. Due to the resistance to apoptosis of cancer cells, photo-pyroptosis can be applied as an alternative strategy for apoptosis induction in cancer treatment. Combining ICB therapy with photo-pyroptosis-related immunotherapy can increase its anti-tumor efficacy further. Therefore, photo-pyroptosis holds promise for cancer treatment, and further research about photo-pyroptosis is necessary.

### Pyroptosis Gets Involved in Photothermal Therapy

PTT is another physical approach for cancer treatment based on nanotechnology that also uses photosensitizers, which can convert light energy into heat when exposed to a specific wavelength of laser irradiation. Previous studies reported the involvement of pyroptosis in the PTT process. Zhao et al. (2020) developed biomimetic nanoparticles loaded with indocyanine green and decitabine for PTT-activated pyroptosis and tumor immunotherapy. Caspase-3 activation and GSDME overexpression were found in the PTT-treated cells (Ploetz et al. 2020; Zhao et al. 2020). Cancer immunotherapy can also be triggered in PTT-induced pyroptosis, further enhancing ICB therapy, similar to photo-pyroptosis of PDT. Zhou et al. (2023) Reported the combination of PTT-triggered pyroptosis and ICB therapy. They developed a multifunctional three-dimensional nanoparticle comprised of gold nanocages, thrombin, and anti-PD-1 antibodies to inhibit tumor growth and recurrence. Under laser irradiation, the nanoparticles exhibited a good photothermal effect and triggered pyroptosis to kill cancer cells. And the anti-PD-1 therapy was enhanced by PTT-induced pyroptosis in their study.

Previous studies have also validated the presence of pyroptosis in the combination therapy of PTT and other methods. Deng et al. (2022) developed a synergistic PTT and chemodynamic therapy (CDT) therapy for treating cancer by coating a metal–organic framework with polydopamine, chemical drug piperlongumine, and photosensitizer indocyanine green derivative-820. They found that caspase-1/GSDMD-mediated pyroptosis is one of the primary forms of programmed cell death in this treatment process, in addition to ferroptosis induced by CDT (Deng et al. 2022). In a study using near infrared (NIR) nanomicelles with combined PDT and PTT characteristics for cancer treatment, caspase-1/GSDMD-dependent pyroptosis was also identified in the treatment process. And it further promoted anti-tumor immune response (Guo et al. 2022). This study confirmed the overall efficacy of PDT and PTT in inducing pyroptosis and promoting anti-tumor immune responses. However, further research is required to determine the specific mechanism of pyroptosis triggered by PTT alone.

## Ultrasound-Based Physical Approaches and Pyroptosis

SDT is another typical nanotechnology-based physical approach that employs ultrasonic waves to treat cancer. It relies on the production of oxidative reactions under low-frequency ultrasound irradiation. Hence, like PDT, SDT may also be associated with pyroptosis and pyroptosis-triggered immunotherapy. Focused ultrasound (FUS) for tumor ablation is the representative method of ultrasound-based physical approaches in cancer treatment. It uses high-intensity ultrasound to exert mechanical and heating effects on tissues. It has been established that FUS may be associated with anti-tumor immune responses. This section discussed SDT-triggered pyroptosis and the possible relationship between pyroptosis and FUS.

### Sonodynamic Therapy-Triggered Pyroptosis Triggers Further Immunotherapy for Cancer Treatment via ROS Production

SDT is a minimally invasive therapeutic approach based on the sonodynamic effect of photosensitizers under the irradiation of ultrasonic beams. The specific mechanism of SDT is still under debate, although its effectiveness in cancer treatment is widely accepted. Similar to PDT, the cell toxicity of SDT results from ROS production during ultrasonic cavitation (Qian et al. 2016). As mentioned above, ROS produced by PDT may result in photo-pyroptosis in the caspase-1/GSDMD pathway. Therefore, it is plausible that ROS-triggered pyroptosis may also occur in SDT, which has been confirmed by several studies on cancer treatment of SDT.

**Fig. 5:**
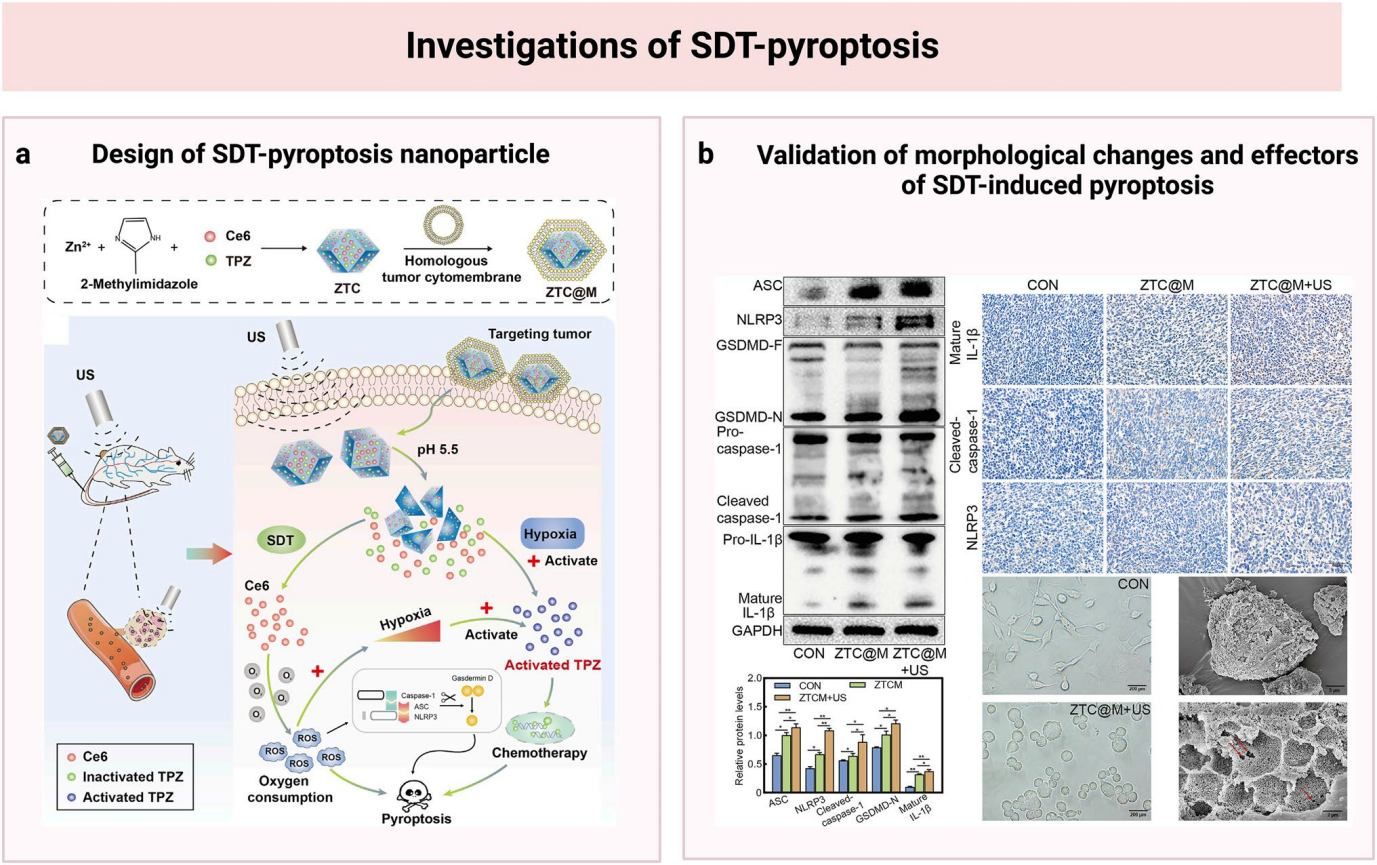
Schematic and validation of sonodynamic therapy (SDT)-induced pyroptosis. **a** The design of SDT-pyroptosis nanoparticle; **b** Validation of morphological changes and effectors of SDT-induced pyroptosis. The sonodynamic nanoparticles were responsive to the low-pH microenvironment of tumors. Chlorin e6 (Ce6) could exert the sonodynamic effect on tumor cells upon irradiation of low-frequency ultrasonic beams by producing cytotoxic reactive oxygen species (ROS). The SDT process consumed oxygen and attenuated hypoxia in cells, enhancing the cell-killing effect of tirapazamine (TPZ). SDT and chemotherapy of TPZ could synergistically trigger pyroptosis of tumor cells. And SDT induced pyroptosis through the caspase-1/GSDMD pathway. The involvement of pyroptosis was verified by detecting cell pyroptotic changes and producing pyroptotic products (Yu et al. 2022). The figure was created with BioRender.com. Printed with permission from Frontiers in Bioengineering and Biotechnology. Copyright 2022, Yu, Cao, Han, Wang, Qiu, Wang, Wei, Wang, Zhang, Liu, Mo and Chen

Several studies that observed cellular morphological changes of pyroptosis in SDT-treated cancer cells suggested the existence of pyroptosis in SDT. The earliest evidence of the role of pyroptosis in the SDT process was discovered by Zhu et al. (2020) in the combination therapy of SDT and PDT. Yang et al. (2020) also noted the pyroptosis morphology in the cancer cells treated with SDT. They utilized ultrasound microbubbles (MBs) loaded with 5-Aminolevulinic acid hydrochloride (ALA) to mediate SDT for treating pancreatic cancer. Typical pyroptosis features, including cellular swelling and microvilli disappearance, were visible in the ALA-MBs-treated groups. Yu et al. (2022) further investigated the mechanism of SDT-induced pyroptosis in their study. The researchers used synergistic therapy of SDT and chemotherapy to treat gastric cancer via zeolitic imidazole frameworks-8 nanoparticles encapsulating hydrophobic Chlorin e6 and hydrophilic tirapazamin and wrapped by cellular membranes of tumor cells. ROS was proved to be the main therapeutic effector of synergistic therapy. The researchers further investigated the involvement of pyroptosis in the ROS-generating SDT process by determining the levels of pyroptosis-related proteins in the treated cells. They found increased cleaved-caspase-1, NLRP3, GSDMD-N, and related inflammatory cytokines in the cells treated with nanoparticles upon low-frequency ultrasound irradiation (Yu et al. 2022). Additionally, pyroptosis-specific cellular morphological features were observed. The schematic of the SDT nanoparticle and validation of pyroptosis is shown in Fig. 5.

The nanoparticles for SDT could also serve as platforms for cancer immunotherapy through pyroptosis induction and subsequent cytokine release. Chen et al. (2023) developed TGF-β1 receptor inhibitor-loaded porous coordination network nanoparticles that could camouflage red blood cell membranes to trigger SDT and extracellular matrix formation for breast cancer treatment. Through the GSDME-caspase-3 pathway, the nanoparticle triggers pyroptosis and a potent immunological response. CTL infiltration was observed in the tumor tissues. Moreover, the researchers validated memory T cell response activation following SDT-triggered pyroptosis treatment. Pyroptotic therapy strongly inhibited the growth of re-challenge tumors in mice and stimulated memory T cell proliferation. This is the first study to show the anti-tumor immunity elicited by SDT-triggered pyroptosis and its efficacy in preventing tumor recurrence (Chen et al. 2023).

Although less investigated than photo-pyroptosis, SDT shared a similar mechanism with PDT in triggering pyroptosis, in which ROS production plays a key role. Additionally, both SDT- and PDT-induced pyroptosis can activate the immune response to improve treatment efficacy. Further research is needed to understand the specific mechanisms of SDT-triggered pyroptosis and its combination with ICB.

### Association Between Pyroptosis and Focused Ultrasound Therapy Needs Further Investigation

High-intensity focused ultrasound (HIFU) is a non-invasive approach that uses high-energy ultrasonic beams to treat solid regional tumors. High acoustic energy concentration can trigger rapid cancer destruction in a localized tumor without damaging adjacent tissues. HIFU ablates tumors via the thermal, mechanical effect, or a combination of the two effects of ultrasonic beams. Previous research has shown that HIFU treatment changes immune systems and activates inflammatory responses, but its association with pyroptosis has not been elucidated yet. DAMPs produced by HIFU may trigger a weak immune response. Additionally, studies on the interaction between HIFU and other stimuli have confirmed that HIFU might trigger potent anti-tumor immunotherapy when combined with other immune methods (Eranki et al. 2020; Fite et al. 2021). It is reported that HIFU-triggered thermal ablation might be unfavorable to immune responses of tumor tissues due to the heat-associated fixation of tumors, which presents a low antigen-releasing rate and little infiltrating immunocytes (Hu et al. 2007; Silvestrini et al. 2017). Hu et al. (2007) found that the HIFU-triggered immune changes, which were more pronounced in the cells treated with mechanical HIFU, may be largely attributed to DC activation. It has also been revealed that histotripsy, a HIFU method that mainly depends on mechanical effect rather than thermal effect, could improve anti-tumoral immune responses (Eranki et al. 2018; Hendricks-Wenger et al. 2021; Vlaisavljevich et al. 2014). However, the role of pyroptosis in HIFU has not yet been fully understood. And the mechanism of HIFU-related immune response and the participation of pyroptosis in HIFU-induced immunotherapy needs further investigation.

## Radiotherapy and Pyroptosis

RT is a crucial treatment option for various cancers in the clinical, including lung cancer, esophageal carcinoma, cervical cancer, and nasopharyngeal carcinoma. High doses of ionizing radiation (IR) used in radiotherapy augment ROS production and cause DNA damage to kill cancer cells. Apoptosis is the classical form of cell death in RT, and it is still unclear whether other types of PCD are also present.

The anti-tumor immune responses following RT have been verified in some tumor models, which showed promising efficacy in suppressing tumors and distant metastasis (Vanpouille-Box et al. 2015). Tumor cells may secret various antigens after radiation, thus activating antigen-specific immune responses (Asano et al. 2011). Ionizing radiation may also release DAMPs to activate DCs and subsequent immunotherapy (Park et al. 2014). Given the intimate association of pyroptosis and immunotherapy, interest in pyroptosis and pyroptosis-triggered immunotherapy in RT has grown.

**Fig. 6:**
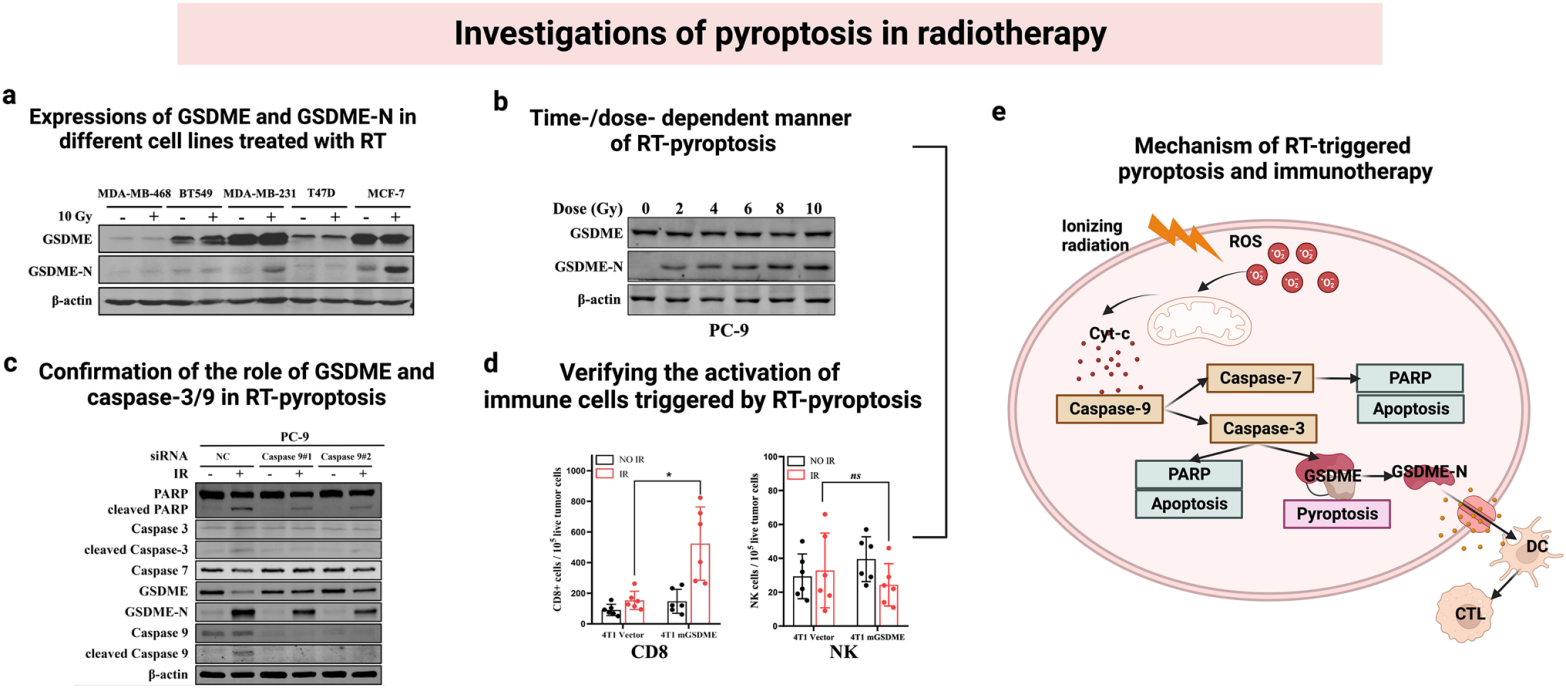
Schematic and validation of radiotherapy (RT)-induced pyroptosis. **a** The expression of GSDME and GSDME-N in different cell lines treated with RT; **b** Time-/dose-dependent manner of RT-pyroptosis; **c** The confirmation of the role of GSDME and caspase-3/9 in RT-pyroptosis; **d** Verifying the activation of immune cells triggered by RT-pyroptosis; **e** The mechanism of RT-triggered pyroptosis and immunotherapy. In cells with high GSDME expression, cleaved GSDME and pyroptotic changes were detected. The authors further revealed RT-induced pyroptosis in tumor cells in a time and dose-dependent manner. They further verified the leading role of GSDME in RT-pyroptosis via the caspase 9/caspase 3 pathway by testing the products of apoptosis and pyroptosis. An increased activation of immune cells was detected, indicating the immunotherapy-triggering effect of RT-pyroptosis (Cao et al. 2023). The figure was created with BioRender.com. Printed with permission from Elsevier. Copyright 2022, Elsevier Inc

According to Cao et al. (2023), RT can trigger pyroptosis in tumor cells through the caspase-9/caspase-3/GSDME pathway in a time-dependent and dose-dependent manner. Their study found that tumor cells with high GSDME expression exhibited typical pyroptosis morphology after receiving RT, including cell swelling and pore formation on plasma membranes. DNA demethylation agents or drugs that increase GSDME expression in tumor cells may enhance pyroptosis in RT treatment. Gene knockdown experiments also proved the involvement of caspase-3, caspase-7, and caspase-9. RT-triggered pyroptosis may also activate anti-tumor immune response through the activation of DCs and CTLs, thus enhancing anti-tumor efficacy. The schematic of the study is shown in Fig. 6. The authors suggested that ROS generation of radiation could activate pyroptosis via GSDME/caspase-9/caspase-3 pathway in addition to apoptosis, which could further activate an anti-tumor immune response. Their findings may suggest a novel approach for optimizing RT by controlling pyroptosis. Tan et al. (2022) also revealed RT-induced caspase-3-dependent pyroptosis in the intestinal epithelial cells. They found that radioresistant colorectal cancer cells had low levels of GSDME, while the adjacent tissues demonstrated high GSDME expressions. The increasing cellular levels of GSDME may sensitize colorectal cancer cells to RT by enhancing pyroptosis. Pyroptosis-triggered anti-tumor immune responses may increase the therapeutic effects of RT. However, RT-triggered pyroptosis in adjacent normal tissues may contribute to RT-related gut damage (Tan et al. 2022). In addition to the gut damage caused by RT-induced pyroptosis, GSDMD-mediated pyroptosis was also related to lethal RT damage in the bone marrow transplantation mouse model (Xiao et al. 2020). Thus, pyroptosis may function in RT as a mechanism for killing cancer cells and as a cause of tissue damage. Future research should focus on increasing RT-triggered pyroptosis in target regions without damaging adjacent normal tissues.

Researchers also revealed the association between radiosensitivity and pyroptosis. MicroRNAs (miRNAs), noncoding RNAs that suppress gene expressions and participate in biological processes, have been found to augment radiation resistance (Shahid et al. 2018; Wang et al. 2013a). Compared to radiosensitive tissues, the radioresistant tumor cells showed reduced pyroptosis and higher levels of miRNA-1290 expression. MiRNA-1290 suppressed RT-induced pyroptosis and decreased the radiosensitivity of triple-negative breast cancer cells by targeting NLRP3. The findings indicated that miRNA-1290 might impair the radiosensitivity of triple-negative breast cancer cells by inhibiting NLRP3-mediated pyroptosis, which might be regarded as a novel therapeutic option (Li and Li 2022). Therefore, the control of pyroptosis can potentially impact the radiosensitivity of cancer cells, which is a target for improving RT treatment of cancer.

## Electricity-Based Physical Approaches and Pyroptosis

### The Participation of Pyroptosis in High-Frequency Irreversible Electroporation (H-FIRE) Is Verified

Irreversible electroporation (IRE) is another ablation method for treating solid tumors-based electroporation phenomemon, in which cellular membrane pores emerge when exposed to high-magnitude electric fields (Rubinsky et al. 2007). High-frequency irreversible electroporation (H-FIRE) is a novel electroporation method as an alternative to conventional IRE, due to its high treatment efficiency and safety (He et al. 2021). Conventional IRE often delivers electric pulses of 70–100 μs, whereas the delivered pulses of H-FIRE are bipolar pulses with 1–5 μs length (Batista Napotnik et al. 2012). Mercadal et al. (2020) investigated the mechanisms and dynamics of cell death in IRE and H-FIRE. The expression of caspase 3/7 was only detected in H-FIRE, indicating that other PCD modes, such as pyroptosis, might coexist in H-FIRE (Mercadal et al. 2020). The existence of pyroptosis was further identified in H-FIRE-treated breast cancer cells by Scaia et al. (2019). Both in vivo and in vitro experiments revealed that cells treated with H-FIRE expressed genes associated with pyroptosis and necroptosis. The study also identified additional immune responses triggered by pyroptosis, which might also contribute to the cancer-killing effect of H-FIRE. The mechanism of H-FIRE-related pyroptosis and its implications for immune responses has not been elucidated yet, and further studies are needed. A more comprehensive design utilizing H-FIRE and pyroptosis-induced immunotherapy may enhance its therapeutic effect in cancers, which warrants further investigations.

### Pyroptosis Is Related to the Systematic Inflammation of Radiofrequency Ablation

Radiofrequency ablation (RFA), an electricity-based minimally invasive method, has been popularized in treating hepatic cancer. An electrical circuit is applied during RFA treatment, generating tumor ablation heat. Although few studies have investigated the relationship between RFA and pyroptosis, some have shown that regulating pyroptosis in RFA may have clinical benefits.

Recent studies reported that endothelial cells can undergo pyroptosis triggered by caspase-1/GSDMD when hepatic hemangiomas are treated with RFA. Inflammatory cytokines, IL-1β and IL-18, were produced during the ablation process, and these cytokines may contribute to systemic inflammatory responses in the patients (Wang et al. 2019; Yang et al. 2019). It has been demonstrated that incomplete RFA causes chemotherapy resistance in hepatocellular carcinoma by affecting the pyroptosis of cancer cells (Wang et al. 2021). Controlling pyroptosis of RFA-treated tissues might be helpful in decreasing the inflammatory side effects of RFA. And the involvement of pyroptosis in the treatment process of RFA and the underlying mechanisms still need further validation.

## Summary and Future Perspectives

Pyroptosis, a newly defined PCD form, has shown its potential in cancer treatment. The inflammasomes and gasdermin protein family are critical effectors of the pyroptosis pathway. Apart from causing cell death, pyroptosis can also induce inflammatory cytokine release, which promotes DC maturation and CTL infiltration, causing a series of immune responses. The cell-killing and immunogenetic nature of pyroptosis makes it a promising strategy in cancer treatment. Physical approaches, an essential component of cancer treatment, including nanotechnology-based physical approaches, RT, and RFA, can trigger pyroptosis via different pathways. The canonical pathway of pyroptosis, involving caspase-1 and GSDMD, has been found in PDT, SDT, and RT-induced pyroptosis. Other non-canonical pathways, such as the caspase-3/caspase-8/GSDME pathway, have also been reported in PDT and RT-induced pyroptosis. Additional research is still needed to uncover the specific mechanisms of pyroptosis in various physical approaches. Pyroptosis can trigger anti-tumor immune responses via inflammatory cytokine release, making it possible to augment treatment efficacy via the synergy of physical approach-induced pyroptosis and immunotherapy. The supplementary immune response might also aid in inhibiting distant tumors. There is increasing research interest in integrating physical approaches with immunotherapy through pyroptosis. Another interesting topic in this sector is the combination of physical approach-triggered pyroptosis and immune checkpoint blockade therapy. The controlling of pyroptosis by adjusting the expressions of pyroptosis-related proteins, such as the gasdermin family, has been explored in previous studies of chemotherapeutic cancer treatment. However, only a few studies have mentioned similar methods in physical approaches-induced pyroptosis (Li et al. 2021; Li and Li 2022), which requires further investigations.

## Conclusion

Pyroptosis is a novel type of PCD that triggers cell death by involving the gasdermin family and inflammasome. It is accompanied by immune responses, due to the release of inflammatory cytokine release, further causing an anti-tumor effect. Physical approaches for treating cancers can trigger pyroptosis, which can further trigger immunotherapy for a synergistic treatment. The efficacy of cancer suppression through physical approaches could be enhanced by manipulating pyroptosis.

## Data Availability

Data sharing not applicable to this article as no datasets were generated or analyzed during the current study.

## Abbreviations

PCD: Programmed cell death
GSDM: Gasdermin
DAMPs: Danger-associated molecular patterns
PAMPs: Pathogen-associated molecular patterns
RT: Radiotherapy
PDT: Photodynamic therapy
PTT: Photothermal therapy
SDT: Sonodynamic therapy
PS: Photosensitizers
ROS: Reactive oxygen species
7-AAD: 7-Aminoactinomycin D
PI: Propidine iodide
GSDMA: Gasdermin A
GSDMB: Gasdermin B
GSDMC: Gasdermin C
GSDMD: Gasdermin D
GSDME: Gasdermin E
DFNB59: Deafness, autosomal recessive 59 protein
PFD: Pore-forming domain
RD: Repressor domain
NOD: Nucleotide-binding oligomerization domain
NLRP1: NOD-like receptor family pyrin domain-containing protein 1
NLRP3: NOD-like receptor family pyrin domain-containing protein 3
CARD: NOD-like receptor family caspase recruitment domain
NLRC4: CARD domain-containing protein 4
AIM2: Absent in melanoma 2
HMGB1: High mobility group protein B1
ATP: Adenosine triphosphate
LPS: Lipopolysaccharide
NK: Natural killer
IFN-γ: Interferon-γ
CTLs: Cytotoxic T lymphocytes
DCs: Dendritic cell
CAIX: Carbonic anhydrase IX
PKM2: Pyruvate kinase M2
IDO: Indoleamine 2,3-dioxygenase
ICB: Immune checkpoint blockade
GSH: Glutathione
1-MT: 1-methyltryptophan
PD-1: Programmed death-1
CDT: Chemodynamic therapy
ALA: Aminolevulinic acid hydrochloride
MBs: Microbubbles
FUS: Focused ultrasound
HIFU: High-intensity focused ultrasound
IR: Ionizing radiation
MiRNAs: MicroRNAs
IRE: Irreversible electroporation
H-FIRE: High-frequency irreversible electroporation
RFA: Radiofrequency ablation
